# Nitrogen-Doped Straw Biochar Reduces Lead Toxicity in Paddy Rhizosphere Soil Through Physicochemical and Microbial Synergies

**DOI:** 10.3390/toxics14070561

**Published:** 2026-06-26

**Authors:** Honghong Li, Zeyu Liu, Zhou Li, Chunle Chen, Meiya Wang

**Affiliations:** 1 School of History and Geography, Minnan Normal University, Zhangzhou 363000, China; 2 Institute of Subtropical Agriculture, Fujian Academy of Agricultural Sciences, Zhangzhou 363005, China; 3 School of Resources and Chemical Engineering, Sanming University, Sanming 365004, China

**Keywords:** lead (Pb) contamination, nitrogen-doped biochar, rhizosphere microbiome, iron plaque, soil health, in situ stabilization

## Abstract

Lead (Pb) is a persistent and highly toxic heavy metal that poses significant ecological and human health risks due to its high bioaccumulation potential. In this study, nitrogen-doped biochar (NBC) was synthesized from straw-derived biochar via ball-milling and ammonium nitrate modification to remediate Pb-contaminated soil. Batch adsorption experiments demonstrated that the adsorption process was best described by the Langmuir isotherm model, indicating monolayer adsorption. X-ray photoelectron spectroscopy (XPS) revealed that Pb(II) immobilization by NBC occurred through multiple mechanisms, primarily precipitation and complexation with hydroxyl and pyrrolic-N functional groups. Subsequent pot experiments confirmed that NBC outperformed pristine biochar (BC) in reducing Pb bioavailability. This superior performance was attributed to the ability of NBC to increase soil pore water pH and significantly decrease soil redox potential (Eh). Moreover, compared to the control, a 5% NBC treatment (NBC2) significantly increased soil organic matter (SOM) by 136.24% while concurrently increasing soil available nitrogen (SAN), phosphorus (SAP), and potassium (SAK) by 46.91%, 75.72%, and 42.79%, respectively. Microbiological analyses indicated that NBC application enhanced soil alpha diversity (Chao1, ACE, and Shannon indices) and enriched beneficial bacterial phyla, such as *Proteobacteria* and *Firmicutes*. Random forest analysis identified the acid-soluble Pb fraction and SOM as the main drivers of bacterial operational taxonomic unit (OTU) composition. Specifically, NBC increased the relative abundance of the family *Hungateiclostridiaceae*, which may promote soil sulfide production and facilitate the precipitation of Pb into highly insoluble forms, further reducing its mobility and toxicity. Collectively, these findings demonstrate that NBC is a promising soil amendment that leverages both physicochemical and microbial pathways to immobilize Pb, mitigate environmental toxicity, and restore soil ecological health.

## 1. Introduction

Anthropogenic activities have led to widespread environmental degradation, with heavy metal contamination of soil emerging as a particularly severe issue. Lead (Pb), a critical industrial resource widely used in the manufacturing of storage batteries, cables, and sheets, has consequently become a priority pollutant requiring stringent regulation in soil environments [1]. Excessive Pb exposure poses severe human health risks, specifically impairing renal, neurological, and immune functions [2]. Its detrimental impact on the brain development of children and adolescents is of particular concern [3,4]. Given the necessity of maintaining agricultural productivity, effective remediation strategies must not only immobilize Pb but also enhance soil fertility and promote overall soil health.

Biochar, a carbon-dense material produced via the oxygen-limited pyrolysis of organic waste, is extensively used to remediate pollutants across various environmental matrices [5,6]. However, its adsorption capacity is highly variable, depending heavily on its physicochemical properties and interactions with specific target pollutants [7]. Furthermore, the efficacy of pristine biochar is often constrained by inherent structural limitations, such as a paucity of surface functional groups and relatively large particle size. These factors restrict its removal efficiency and limit its application for treating high concentrations of pollutants [8,9]. Consequently, various modification techniques, including chemical and physical modification, impregnation with mineral sorbents, and magnetization, have been employed to enhance the adsorption capacity of biochar [10].

Nitrogen (N) is an essential nutrient; however, straw-derived biochar typically contains limited N, necessitating supplemental fertilization in agricultural applications. Unfortunately, the excessive application of N fertilizers can cause soil acidification, which mobilizes heavy metals and increases their bioavailability and toxicity [11,12]. To address this issue, N-doping has emerged as an effective modification strategy. Previous research indicates that N-doping increases surface sorption sites, augments basicity, and introduces positive surface charges, thereby significantly enhancing pollutant adsorption [13,14]. Furthermore, N-doping improves the overall electrochemical performance of biochar by altering its spin and electronic characteristics [15,16]. N-doped biochar (NBC) can be synthesized either through the pyrolysis of N-rich biomasses or via post-pyrolytic surface modification using compounds such as ammonia, lithium nitrate, aniline, urea, and melamine [17]. The structure of the nitrogenous precursor markedly influences N speciation in the resulting biochar, which subsequently dictates its properties. For instance, urea-modified biochar exhibits a higher proportion of graphitic N (26.6%), whereas melamine-modified biochar contains greater percentages of pyrrolic (35.2%) and pyridinic (36.8%) N [18]. Additionally, NBC exhibits enhanced performance in soil remediation by effectively reducing heavy metal bioavailability and improving nutrient retention [19].

Beyond chemical doping, the adsorptive capacity of biochar can be improved by modifying its particle size and surface morphology. Ball milling is a promising, environmentally friendly physical modification method that is highly adaptable and suitable for large-scale production [20,21]. This process significantly increases the surface area and pore volume of biochar, thereby improving its adsorption capacity for both inorganic and organic contaminants [7]. For example, a micro-nano-engineered nitrogenous bone biochar synthesized via ball milling exhibited a 593.2% increase in specific surface area, which augmented its Pb(II) adsorption capacity by 64.61% compared to pristine biochar [9]. Therefore, combining N-doping with ball milling to produce N-doped microporous biochar presents a highly promising strategy for remediating Pb-contaminated soils while simultaneously supporting agricultural productivity.

Rice (*Oryza sativa* L.) is a major staple crop predominantly cultivated in the southern regions of China, where the soils are primarily acidic. This acidity enhances the bioavailability of heavy metals, thereby elevating the risk of Pb accumulation in rice plants. For instance, an analysis of over 250 rice samples from southeastern China reported grain Pb concentrations of up to 1.136 μg·g^−1^ [22]. Similarly, a study examining rice from contaminated fields in Hunan Province found median Pb concentrations ranging from 0.051 to 0.784 μg·g^−1^ [23]. As rice is typically cultivated in flooded environments, soil oxygen is rapidly depleted, creating highly reducing conditions. However, the release of O_2_ and other oxidants by rice roots oxidizes ferrous iron to ferric iron in the rhizosphere. This results in the precipitation of iron oxides and hydroxides on the root surface, forming an iron plaque [24]. This plaque significantly influences metal bioavailability; the abundant functional groups on iron hydroxides effectively sequester metals through adsorption and co-precipitation, thereby altering elemental uptake and accumulation by the plant [24,25]. Consequently, the bioavailability of heavy metals in the rhizosphere exerts a more direct impact on plant uptake than it does in bulk soil.

The objective of soil remediation extends beyond merely reducing heavy metal bioavailability; it also encompasses the restoration of overall soil ecological function. Rhizosphere bacteria are highly sensitive to environmental stressors [26]. Consequently, indicators such as microbial biomass, enzyme activity, and diversity are increasingly utilized to assess soil health [27,28]. Furthermore, these microorganisms drive critical biogeochemical processes; they can adsorb metal ions, mobilize bound heavy metals, facilitate symbiotic N fixation, and stimulate phytohormone release, all of which promote plant development [29,30,31]. Thus, monitoring the rhizosphere microbial community structure is essential for understanding the mechanisms by which NBC remediates heavy metal-contaminated soils. We hypothesized that NBC not only reduces Pb bioavailability but also enhances soil fertility and positively alters the microbial community structure. To test this hypothesis, this study aimed to (1) evaluate the efficacy of NBC in reducing Pb bioavailability and toxicity in rhizosphere soil; (2) assess its impact on microbial biomass and diversity; and (3) elucidate the mechanisms by which NBC facilitates the remediation of Pb-contaminated soils.

## 2. Materials and Methods

### 2.1. Soil and Biochar Preparation

Surface soil samples (0–20 cm) were collected from a heavy metal-polluted farmland in Sanming City, Fujian Province, China (118°15′ E, 26°14′ N). The soil is characterized as sandy clay loam with a pH of 6.50, an organic matter content of 22.0 g kg^−1^, and a total Pb concentration of approximately 2640.0 mg kg^−1^. These elevated Pb levels are primarily attributed to nearby Pb and zinc (Zn) mining activities. The collected samples were air-dried and ground to pass through a 2 mm sieve. The detailed physicochemical properties of the soil are summarized in Table S1.

Pristine corn (*Zea mays* L.) straw biochar (BC), pyrolyzed in a furnace under a N_2_ atmosphere at 500 °C for 2.5 h, was purchased from Lize Environmental Protection Company (Zhengzhou, China). The material was ground to a particle size of <2 mm, thoroughly rinsed with deionized water, and stored under dry conditions until use. The NBC was synthesized following the protocol described by Kasera et al. [18]. Briefly, the pristine BC was ball-milled and sieved through a 0.149 mm nylon mesh. A 10 g sample of the milled BC was then dispersed in 50 mL of deionized water. Ammonium nitrate (NH_4_NO_3_) powder (2.85 g) was added to the suspension, followed by continuous stirring for 24 h. The mixture was subsequently filtered, and the recovered biochar was dried at 70 °C. The physicochemical properties of the prepared biochars are detailed in Table S2.

### 2.2. Adsorption Experiments

Batch adsorption experiments were conducted to evaluate the Pb(II) adsorption capacity of NBC. For the kinetic studies, NBC (1.25 g·L^−1^) was suspended in a 200 mg·L^−1^ Pb(II) solution (pH 5.0) and agitated at 180 rpm at 25 ± 2 °C. Aliquots were withdrawn at predetermined intervals over a 48 h period. All experiments were performed in triplicate. Prior to analysis, the samples were filtered through a 0.22 μm Millipore filter (Merck, Darmstadt, Germany), and the residual Pb(II) concentrations were determined via flame atomic absorption spectrometry (AAS, PinAAcle 900, Perkin Elmer, Waltham, MA, USA). Adsorption kinetics were evaluated using the nonlinear forms of the pseudo-first-order (Equation (1)) and pseudo-second-order (Equation (2)) models, expressed as follows:

(1) $$q_{t}\;=\;q_{e}\left(1\;-\;e^{-k_{1}t}\right)$$

(2) $$q_{t}=\frac{k_{2}q_{e}^{2}t}{1+k_{2}q_{e}t}$$

where *q_e_* and *q_t_* (mg·g^−1^) represent the adsorption capabilities at equilibrium and at time *t*, respectively, and *k*_1_ (1·min^−1^) and *k*_2_ (g·mg^−1^·min^−1^) are the rate constants for the pseudo-first-order and pseudo-second-order models, respectively.

For the adsorption isotherm experiments, a fixed dosage of NBC (1.25 g·L^−1^) was added to aqueous Pb(II) solutions with varying initial concentrations (0.1–500 mg·L^−1^). The mixtures were maintained at pH 5.0 and agitated at 180 rpm and 25 ± 2 °C. All experiments were performed in triplicate. Samples were collected after a 24 h equilibrium period. The adsorption isotherm data were fitted to the Langmuir (Equation (3)) and Freundlich (Equation (4)) models:

(3) $$q_{e}\;=\;\frac{q_{max}K_{L}C_{e}}{1\;+\;K_{L}C_{e}}$$

(4) $$q_{e}=K_{F}C_{e}^{1/n}$$

where *C_e_* (mg·L^−1^) is the residual Pb(II) concentration at equilibrium; *q_max_* (mg·g^−1^) represents the maximum adsorption capacity; *K_L_* (L·mg^−1^) and *K_F_* (L·g^−1^) are the rate isotherm constants for the Langmuir and Freundlich models, respectively; and *n* is a dimensionless parameter indicating the degree of nonlinearity between the solution concentration and adsorption.

Additionally, the surface chemical properties of the NBC before and after Pb(II) sorption were characterized using X-ray photoelectron spectroscopy (XPS, ESCALAB 250 XI, Thermo Fisher Scientific, Waltham, MA, USA).

### 2.3. Rhizobox Experiments

#### 2.3.1. Experimental Setup

A rhizobox experiment was designed to evaluate the effects of NBC on Pb distribution in the soil solution at varying distances from the rhizosphere. Each rhizobox consisted of a cylindrical container (18 cm in diameter, 15.2 cm in height). A 300-mesh nylon root bag (10 cm in diameter, 15 cm in height) was positioned at the center of the container to separate the rhizosphere from the bulk soil. The mesh size allowed for the exchange of nutrients, metabolites, and microbes while preventing root penetration. Five experimental treatments were established in triplicate: CK (control, no amendment), BC1 (2.5% BC), BC2 (5% BC), NBC1 (2.5% NBC), and NBC2 (5% NBC). The soil and biochar were thoroughly mixed, and 1000 g of the mixture was added to each rhizobox, ensuring the soil level inside the root bag remained consistent with the surrounding soil. Soil moisture was adjusted to 70% of its maximum water-holding capacity and allowed to equilibrate for one week. Subsequently, two rice seedlings (*Oryza sativa* L. ssp. *indica* ‘Jingliangyouhuazhan’) at the three-leaf stage were transplanted into the center of each rhizobox. Throughout the experiment, a 2 cm layer of standing water was maintained to simulate typical paddy growing conditions.

#### 2.3.2. Sample Collection

Rhizon samplers (Rhizosphere Research Products, Wageningen, The Netherlands) were installed within the root bags, as well as at distances of 0.2, 1.0, and 3.0 cm from the rhizosphere boundary. Soil solutions were sampled 30 days after transplanting. Immediately after collection, the samples were filtered. A portion of each filtrate was stored at 4 °C, while the remainder was acidified with 0.1 mL of 0.1 M HNO_3_ to ensure stability for subsequent elemental analysis.

The Pb concentration in the soil solution was quantified using inductively coupled plasma mass spectrometry (ICP-MS, Agilent 7500cx; Agilent Technologies, Santa Clara, CA, USA). Solution pH and electrical conductivity (EC) were measured using a Starer 3100 pH meter (OHAUS, Changzhou, China) and a DDS-308A conductivity meter (Leici, Shanghai, China), respectively. The redox potential (Eh) of both the rhizosphere and bulk soils was determined using an FJA-6 oxidation-reduction meter (ChuanDi, Nangjing, China).

Forty days after transplanting, fresh rhizosphere soil was collected and immediately stored at −80 °C for subsequent bacterial community analysis. The remaining rhizosphere soil and the bulk soil samples were air-dried and passed through a 2 mm nylon mesh sieve. A subset of these air-dried soils was finely ground using an agate mortar and passed through a 0.149 mm nylon sieve. Finally, fresh root samples were thoroughly rinsed in preparation for iron plaque extraction.

#### 2.3.3. Soil Characterization

Soil properties were analyzed according to the methods described by Jackson [32]. Soil pH was measured at a soil/water ratio of 1:5 (*w*/*v*) using a pH meter (Starer 3100, OHAUS, Changzhou, China). Soil organic matter (SOM) content was determined by wet oxidation with potassium dichromate (K_2_Cr_2_O_2_). Soil available phosphorus (SAP) was extracted with a 0.5 M sodium bicarbonate (NaHCO_3_) solution (pH 8.5) and quantified via the molybdenum blue method, with absorbance measured at 700 nm using a spectrophotometer. Soil available potassium (SAK) was extracted with 1 M ammonium acetate (CH_3_COONH_4_) and measured using flame photometry. Alkaline hydrolysable nitrogen (SAN) was determined using the alkaline diffusion method.

Soil available Pb was extracted by shaking 5 g of air-dried soil (<2 mm) with 25 mL of 0.1 M CaCl_2_ solution for 2 h at 25 °C, followed by immediate filtration. To assess Pb fractionation, a sequential extraction procedure based on the modified BCR (Community Bureau of Reference) protocol was employed [33]. This method quantifies four operationally defined fractions: acid-soluble (F1), reducible (F2), oxidizable (F3), and residual (F4). The Pb concentrations in all extracts were determined using ICP-MS (Agilent 7500cx, Agilent Technologies, Santa Clara, CA, USA).

#### 2.3.4. Iron Plaque Extraction and Root Analysis

An iron plaque was extracted from rice roots using the dithionite–citrate–bicarbonate (DCB) method [34]. Washed fresh roots were mixed with 40 mL of DCB solution (0.03 M sodium citrate, 0.125 M sodium bicarbonate, and 0.06 M sodium dithionite) and agitated for 60 min to detach the plaque from the root surfaces. The resulting suspension was filtered, and the filtrate was analyzed for Fe and Pb concentration by ICP-MS. The roots were subsequently rinsed with deionized water and dried at 70 °C for 48 h. Ground subsamples of the dried roots were microwave-digested in a mixture of nitric acid and hydrogen peroxide, after which root Pb concentrations were measured via ICP-MS. Quality assurance and quality control (QA/QC) for the plant Pb analysis were conducted using a certified reference material (GBW-07605; China Standard Materials Research Center, Beijing, China), yielding Pb recovery rates between 90% and 110%. Reagent blanks and analytical duplicates were included in all sample batches to ensure analytical accuracy and precision.

#### 2.3.5. DNA Extraction, Amplification, and Sequencing

Total soil DNA was extracted using a commercial DNA isolation kit (Sangon Biotech, Shanghai, China). The full-length bacterial 16S rRNA gene was amplified using the barcoded conserved primers 27F (5′-AGRGTTTGATYNTGGCTCAG-3′) and 1492R (5′-TASGGHTACCTTGTTASGACTT-3′). The resulting amplicon libraries were sequenced on the PacBio SMRT RS II DNA sequencing platform (Pacific Biosciences, Menlo Park, CA, USA). Raw subreads were processed into circular consensus sequencing (CCS) reads using SMRT Link v8.0 (minPasses ≥ 5; minPredictedAccuracy ≥ 0.9). Chimeric sequences were subsequently removed using UCHIME. The quality-filtered sequences were clustered into operational taxonomic units (OTUs) at a 97% similarity threshold using USEARCH v10.0, and OTUs representing less than 0.005% of the total sequences were discarded. Taxonomic assignment was performed using the naive Bayes classifier (classify-sklearn) in QIIME2 (v2020.6) against the SILVA database (Release 138) with a confidence threshold of 0.7. All bioinformatics analyses were conducted on the BMKCloud platform (Biomarker Technologies, Beijing, China).

### 2.4. Statistical Analysis

Data are expressed as the mean ± standard error (S.E.). Statistical analyses were performed using SPSS 20.0 (IBM, Armonk, NY, USA). Prior to analysis, the normality of residuals and homogeneity of variances were verified using the Shapiro–Wilk and Levene tests, respectively. One-way analysis of variance (ANOVA) was employed for datasets with a single independent variable, whereas two-way ANOVA was used to evaluate main effects and interactions for datasets with factorial structures. Post hoc comparisons were conducted using Duncan’s multiple range test, with statistical significance defined as *p* < 0.05. Mantel tests were conducted to evaluate Spearman’s rank correlations between distance matrices using the “vegan” package in R (v3.5.0) [35]. Microbial community composition was characterized based on phyla with an average relative abundance greater than 0.5% [31]. Alpha diversity of the microbial communities was assessed using the Chao1 and Shannon indices. The most influential environmental factors affecting operational taxonomic units (OTUs) were identified via Random Forest (RF) analysis using the “rfPermute” package in R. All graphs were generated using Origin 2024 (OriginLab, Northampton, MA, USA).

## 3. Results

### 3.1. Pb(II) Adsorption by NBC

The adsorption of NBC exhibited a rapid initial phase, reaching equilibrium within 200 min (Figure S1a). Kinetic data were evaluated using pseudo-first-order and pseudo-second-order models; the experimental results correlated more strongly with the pseudo-second-order model (Table S3), indicating a chemisorption-dominated mechanism [36]. Furthermore, the adsorption capacity of NBC increased progressively with rising equilibrium Pb(II) concentrations (Figure S1b). The Langmuir isotherm model provided the best fit for the experimental data, suggesting that Pb(II) uptake predominantly occurs via monolayer coverage [37]. Based on the Langmuir model, the calculated maximum adsorption capacity was 148.25 mg·g^−1^ (Table S4).

**Figure 1:**
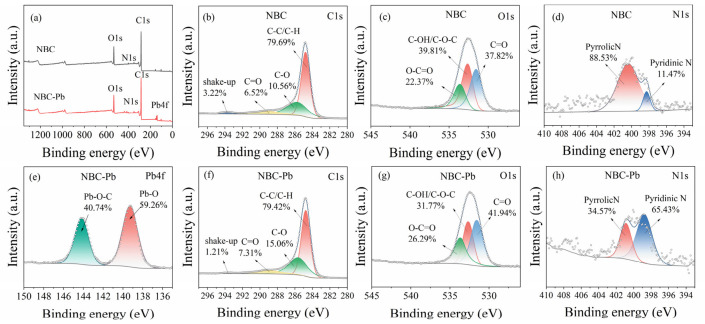
XPS spectra of NBC before and after Pb^2+^ adsorption: (**a**) Survey spectra. (**b**–**d**) High-resolution C1s, O1s, and N1s spectra of pristine NBC. (**e**–**h**) High-resolution Pb4f, C1s, O1s, and N1s spectra of NBC following Pb^2+^ adsorption.

To elucidate the mechanism of Pb(II) adsorption onto NBC, XPS was conducted to analyze the elemental composition and chemical states of the material before and after adsorption. The XPS survey spectrum of the Pb(II)-loaded biochar (NBC-Pb) revealed a distinct Pb4f peak (Figure 1a), confirming successful adsorption. The C1s spectra (Figure 1b,f) showed that the C-C/C-H peak ratio remained stable, indicating that the carbon skeleton was largely unaffected by the adsorption process. However, following absorption, the relative proportions of C-O increased from 10.56% (286.28 eV) in pristine NBC to 15.06% (286.48 eV) in NBC-Pb, while the proportion of C=O increased from 6.25% (287.58 eV) to 7.31% (288.98 eV). Additionally, the O1s spectra (Figure 1c,g) revealed an increase in O-C=O proportion from 22.37% (533.60 eV) to 26.29% (533.64 eV) following adsorption, suggesting the formation of lead carbonate precipitates [38]. Conversely, the C-OH/C-O-C proportion decreased from 39.81% (532.61 eV) to 31.77% (532.64 eV), demonstrating the participation of surface hydroxyl groups in Pb(II) binding.

Regarding nitrogen speciation, nitrogen in NBC generally exists as chemically bonded N and structural N, with the latter directly incorporated into the carbon skeleton [39]. In this study, structural N in pristine NBC was identified as pyrrolic N (400.24 eV) and pyridinic N (398.28 eV). Following Pb(II) adsorption, the proportion of pyrrolic N decreased substantially from 88.53% to 34.57% (400.88 eV), confirming its active involvement in Pb(II) binding. This aligns with previous studies indicating that pyrrolic N plays a more critical role than other structural N forms in the adsorption of heavy metals, such as Cd [40].

### 3.2. Spatial Distribution of pH, EC, Eh and Pb in Rhizosphere

**Figure 2:**
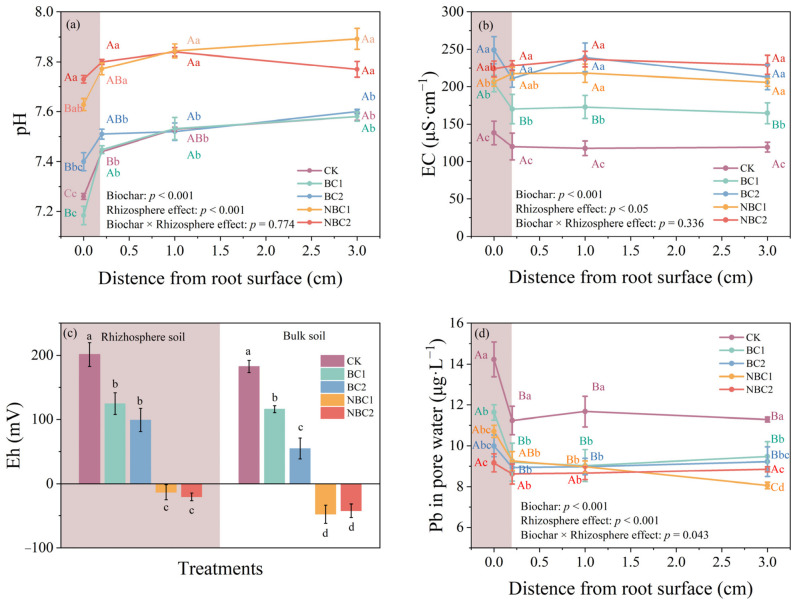
Spatial distribution of (**a**) pH, (**b**) EC, (**c**) Eh, and (**d**) Pb in soil pore water. Values represent the mean ± SE (*n* = 3). Different lowercase letters indicate significant differences among treatments within the same soil compartment, whereas different uppercase letters indicate significant differences across varying distances from the root within the same treatment. Statistical significance was evaluated using a two-way ANOVA followed by Duncan’s multiple-range test (*p* < 0.05). Treatments: CK, control (no amendment); BC1, 2.5% straw biochar; BC2, 5% straw biochar; NBC1, 2.5% N-doped straw biochar; NBC2, 5% N-doped straw biochar.

The pH of the rhizosphere pore water was significantly lower than that of the bulk soil (Figure 2a). Across the treatments, the rhizosphere pore water pH followed the order NBC2 > NBC1 > BC2 > CK > BC1. Notably, the application of NBC increased the pore water pH to a greater extent than unmodified BC. The electrical conductivity (EC) of the soil pore water increased with biochar addition (Figure 2b). Specifically, under the CK, BC1, and BC2 treatments, the EC was higher in the rhizosphere than in the bulk soil.

The redox potential (Eh) of rhizosphere soil was generally higher than that of the bulk soil; however, biochar addition significantly reduced the Eh in both soil zones. Relative to the control, the magnitude of Eh reduction in the rhizosphere followed the order NBC2 (110.24%) > NBC1 (106.60%) > BC2 (50.74%) > BC1 (38.04%) (Figure 2c). Furthermore, the application of either BC or NBC significantly reduced pore water Pb concentration in both the rhizosphere and bulk soils. Compared to the control, rhizosphere pore water Pb concentrations decreased by 18.27%, 29.81%, 24.84%, and 35.64% under the BC1, BC2, NBC1, and NBC2 treatments, respectively. Although pore water Pb concentration remained consistently higher in the rhizosphere than in the bulk soil, the relative magnitude of this rhizosphere enrichment varied across treatments as follows: NBC1 (30.89%) > CK (26.74%) > BC1 (22.84%) > BC2 (8.32%) > NBC2 (3.55%).

### 3.3. Effects of N-Doped Straw Biochar on Soil Properties

**Figure 3:**
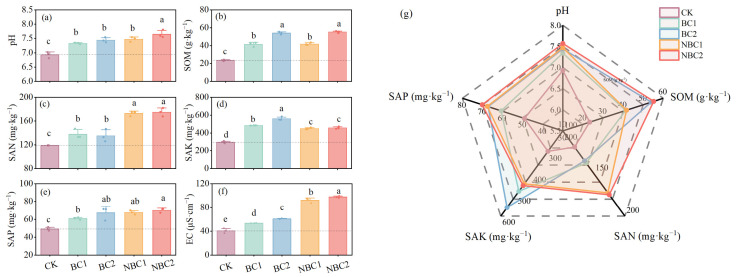
Rhizosphere soil characteristics under N-doped straw biochar treatment: (**a**–**f**) Individual plots for pH, SOM, SAN, SAK, SAP, and EC. (**g**) Radar chart comparing the soil nutrient indicators. Values are presented as the mean ± SE. Different lowercase letters above the bars indicate significant differences among the treatments (*p* < 0.05). Abbreviations: SOM, soil organic matter; SAN, soil available nitrogen; SAK, soil available potassium; SAP, soil available phosphate. Treatments: CK, control (no amendment); BC1, 2.5% straw biochar; BC2, 5% straw biochar; NBC1, 2.5% N-doped straw biochar; NBC2, 5% N-doped straw biochar.

Biochar application significantly improved soil properties (Figure 3). The addition of NBC produced a substantially greater increase in SAN than unmodified BC. Compared to the control, BC increased SAN by 13.50–15.67%, whereas NBC yielded a significantly higher increase of 45.04–46.91%. Conversely, BC was more effective than NBC at enhancing SAK, increasing it by 86.12–115.42% relative to the control, compared to the 72.53–75.72% increase observed with NBC. Overall, the NBC2 treatment resulted in the most pronounced improvements; compared to the control, it significantly increased soil pH, EC, SOM, SAN, SAK, and SAP by 9.29%, 7.23%, 136.24%, 46.91%, 75.72%, and 42.79%, respectively.

### 3.4. Pb Availability and BCR Sequential Extraction in Soil

**Figure 4:**
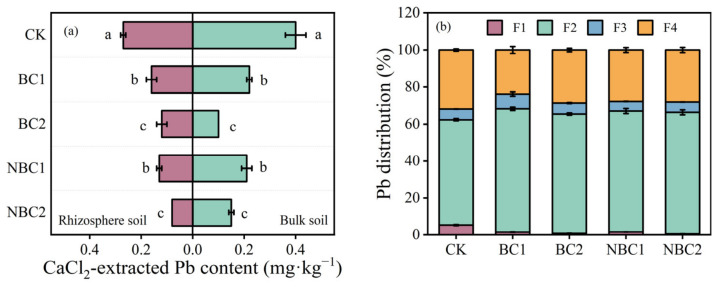
Effects of biochar treatments on (**a**) CaCl_2_-extracted Pb content in soils and (**b**) Pb fractions in rhizosphere soil, as determined by the BCR sequential extraction procedure. Values represent means ± SE. Different lowercase letters indicate significant differences among treatments (*p* < 0.05). Pb fractions: F1, acid-soluble; F2, reducible; F3, oxidizable; F4, residual. Treatments: CK, control (no amendment); BC1, 2.5% straw biochar; BC2, 5% straw biochar; NBC1, 2.5% N-doped straw biochar; NBC2, 5% N-doped straw biochar.

The application of both BC and NBC effectively decreased the bioavailability of Pb in the rhizosphere and bulk soils. Notably, increasing the biochar application rate significantly reduced the CaCl_2_-extractable Pb content in the soils (Figure 4a). Furthermore, CaCl_2_-extractable Pb concentrations were consistently lower in the rhizosphere than in the bulk soil. Compared to the control, biochar application reduced CaCl_2_-extracted Pb in the rhizosphere in the following descending order: NBC2 (68.77%) > BC2 (55.06%) > NBC1 (50.25%) > BC1 (42.08%).

Additionally, soil Pb fractions were analyzed using BCR sequential extraction. In the control treatment, the distribution of Pb across the acid-soluble, reducible, oxidizable, and residual fractions was 5.24%, 57.10%, 5.69%, and 31.96%, respectively (Figure 4b). Biochar application decreased the proportion of acid-soluble Pb while concomitantly increasing the reducible Pb fraction.

### 3.5. Iron Plaque and Pb Accumulation in Rice Roots

**Figure 5:**
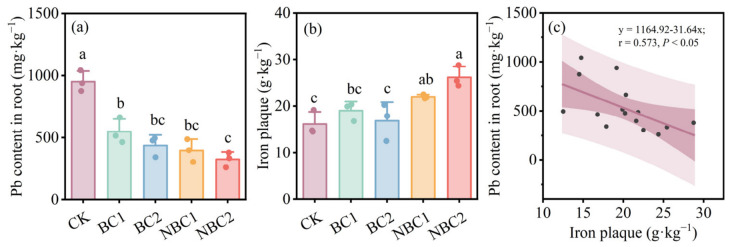
(**a**) The concentration of Pb in rice roots, (**b**) the amount of iron plaque on rice roots, and (**c**) the correlation between these two factors. Values represent means ± SE. Different lowercase letters above the bars indicate significant differences among the treatments (*p* < 0.05). Treatments: CK, control (no amendment); BC1, 2.5% straw biochar; BC2, 5% straw biochar; NBC1, 2.5% N-doped straw biochar; NBC2, 5% N-doped straw biochar. Treatments: CK, control (no amendment); BC1, 2.5% straw biochar; BC2, 5% straw biochar; NBC1, 2.5% N-doped straw biochar; NBC2, 5% N-doped straw biochar.

The addition of either BC or NBC significantly reduced the Pb content in rice roots (Figure 5a). Compared to the control, the reduction in root Pb followed the order NBC2 (65.97%) > NBC1 (58.38%) > BC2 (54.04%) > BC1 (42.47%). While the BC application had no significant effect on the formation of iron plaque on the root surface, the NBC application significantly increased it by 21.98–26.19%. Furthermore, a significant negative correlation was observed between the amount of root iron plaque and root Pb content (*p* < 0.05).

### 3.6. Bacterial Community Composition

**Figure 6:**
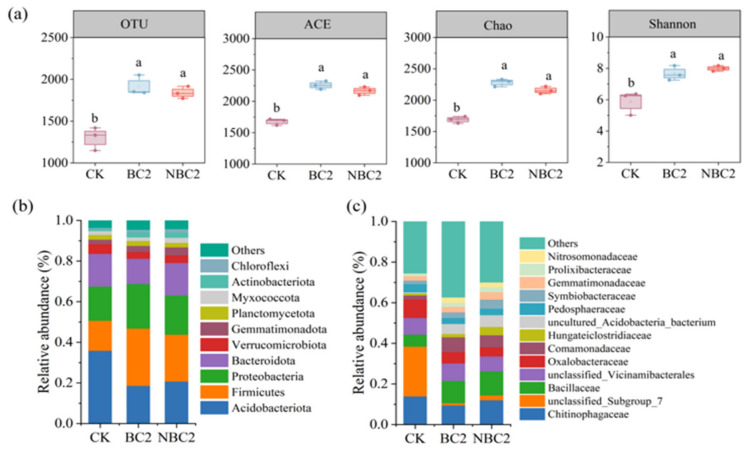
(**a**) Bacterial alpha diversity indices and (**b**) the dominant bacterial community composition at the phylum and (**c**) family levels. Different lowercase letters indicate significant differences among the treatments (*p* < 0.05). Treatments: CK, control (no amendment); BC2, 5% straw biochar; NBC2, 5% N-doped straw biochar.

Biochar application significantly affected soil microbial diversity (Figure 6a). Compared with the control group (CK), the number of OUTs, as well as the ACE, Chao1, and Shannon indices, significantly increased in the BC2 and NBC2 treatments (*p* < 0.05). This indicates that the higher application rates of both biochars (BC2 and NBC2) substantially enhanced the richness and diversity of bacterial communities in the Pb-contaminated soil.

At the phylum level, the dominant phyla were *Acidobacteriota*, *Proteobacteria*, *Firmicutes*, *Bacteroidota*, and *Gemmatimonadota* (Figure 6b). Relative to CK, the BC2 and NBC2 treatments significantly increased the relative abundances of *Proteobacteria*, *Firmicutes*, and *Actinobacteriota*, whereas *Acidobacteriota* exhibited a corresponding decline. These findings suggest that biochar application selectively enriches or inhibits specific bacterial phyla. At the family level, *Comamonadaceae*, *Oxalobacteraceae*, *Bacillaceae*, and *Chitinophagaceae* were the predominant taxa. The relative abundances of *Bacillaceae*, *Comamonadaceae*, and *Nitrosomonadaceae* were significantly higher in the BC2 and NBC2 treatments than in the CK, whereas *Oxalobacteraceae* exhibited the opposite trend. Additionally, the NBC2 treatment resulted in a greater relative abundance of *Hungateiclostridiaceae* and *Symbiobacteraceae* compared to the BC2 treatment (Figure 6c).

### 3.7. Relationships Between Environmental Factors and Bacterial Characteristics

**Figure 7:**
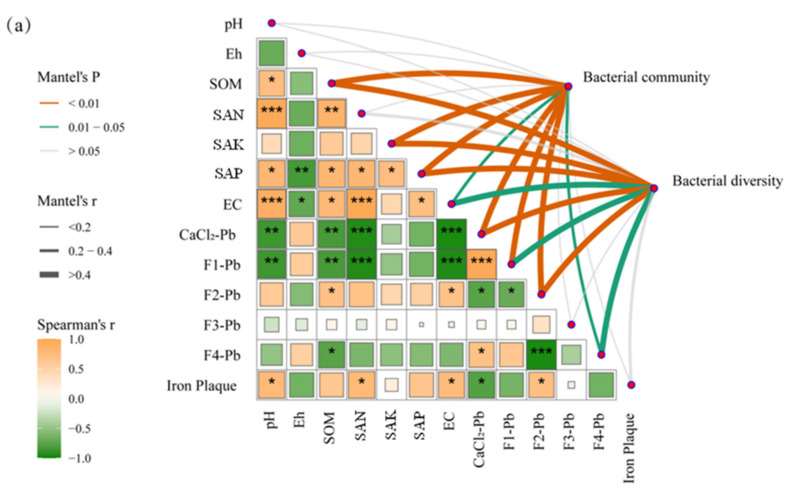

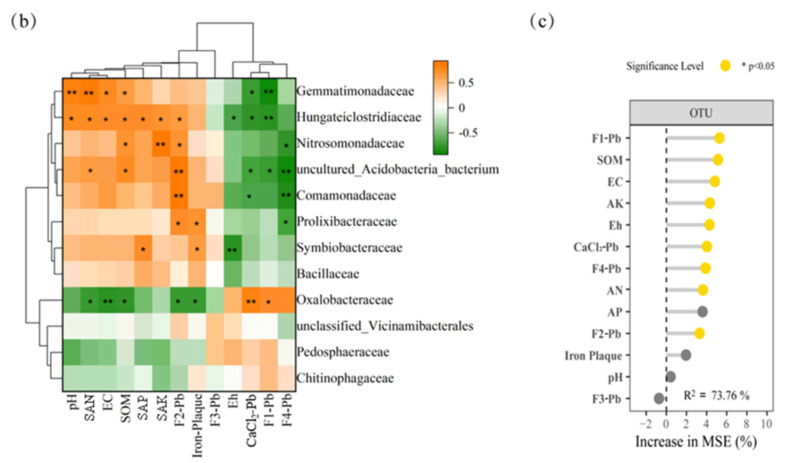
Relationships between bacterial communities and environmental factors: (**a**) Correlations between bacterial community composition, diversity, and environmental factors. Line width is proportional to the partial Mantel’s *r* value, and line color denotes statistical significance (orange, *p* < 0.001; blue, 0.001 ≤ *p* < 0.01; green, 0.01 ≤ *p* < 0.05; grey, *p* ≥ 0.05). All *p*-values were adjusted for multiple testing using the Bonferroni–Holm method. (**b**) Spearman correlation heatmap between environmental factors and individual bacterial families. *, ** and *** indicate significance at *p* < 0.05, *p* < 0.01 and *p* < 0.001, respectively. (**c**) Relative importance of environmental factors in predicting OTUs, as assessed by a random forest model. Abbreviations: EC, electrical conductivity; Eh, redox potential; SOM, soil organic matter; SAN, soil available nitrogen; SAK, soil available potassium; SAP, soil available phosphate; CaCl_2_-Pb, CaCl_2_-extractable Pb; F1-Pb, acid-soluble Pb fraction; F2-Pb, reducible Pb fraction; F3-Pb, oxidizable Pb fraction; F4-Pb, residual Pb fraction.

The bacterial community composition and diversity were significantly correlated with several environmental factors, including SOM, SAK, SAP, CaCl_2_-Pb, EC, F1-Pb, F2-Pb, and F4-Pb (*p* < 0.05, Figure 7a). To further evaluate the influence of these factors on bacterial OTUs, a random forest (RF) analysis was performed (Figure 7c). The F1-Pb emerged as the most important predictor, followed by SOM and EC, highlighting the prominent role of bioavailable Pb in shaping the soil bacterial community structure. To assess the effects of environmental variables on specific soil microbial taxa, their relationships with the relative abundance of individual bacterial families were analyzed (Figure 7b). As anaerobic taxa, *Hungateiclostridiaceae* and *Symbiobacteraceae* exhibited significant negative correlations with soil Eh. Furthermore, the relative abundance of *Gemmatimonadaceae* was positively correlated with pH, SAN, EC, and SOM but negatively correlated with CaCl_2_-Pb and F1-Pb. Conversely, *Oxalobacteraceae* demonstrated the exact opposite trend.

## 4. Discussion

### 4.1. Effects of NBC on the Spatial Distribution of Pb in the Rhizosphere Soil

The rhizosphere is a critical zone for nutrient cycling and microbial processes, where heavy metal speciation directly governs plant uptake and subsequent accumulation [27]. In soil solutions, lead (Pb) exists in both cationic (e.g., Pb^2+^, PbCl^+^, PbOH^+^) and anionic (e.g., PbCl_3_^−^ and Pb(CO_3_)_2_^2−^) forms [41]. The solubility of these species facilitates the vertical translocation of Pb from upper soil horizons to lower depths, posing a significant risk of groundwater contamination. Furthermore, the high bioavailability of these species makes Pb readily accessible for plant absorption. In the present study, pore water Pb concentrations were consistently higher in the rhizosphere than in the bulk soil. However, the application of NBC significantly reduced Pb concentrations in the rhizosphere pore water (Figure 2). This reduction can be attributed to three primary mechanisms.

First, the NBC application increased the pH of the soil pore water more effectively than both the BC and control treatments. Typically, rhizosphere pore water exhibits a lower pH than bulk soil because plant roots excrete H^+^ ions to balance the disproportionate uptake of cations over anions [42]; additionally, rice roots specifically secrete organic acids such as acetate and oxalate [43]. By significantly elevating the pore water pH in both the rhizosphere and the bulk soil, NBC substantially decreases Pb solubility. This pH elevation promotes Pb precipitation as hydroxides, phosphates, or carbonates and facilitates the formation of highly stable Pb–organic complexes [41].

Second, the NBC application significantly reduced the soil redox potential (Eh). This effect is likely attributable to the substantial increase in soil nitrogen content induced by NBC compared to BC (Figure 3). An optimized C/N ratio stimulates the microbial decomposition of organic matter [44,45,46], a process that consumes soil oxygen and consequently lowers the redox potential. Under these anaerobic conditions, sulfate (SO_4_^2−^) is reduced to sulfide (S^2−^). In the pore water of these anoxic environments, the resulting sulfide reacts with trace metals to form insoluble metal–sulfide complexes, effectively restricting Pb solubility and mobility [47,48].

Third, batch experiments demonstrated that the NBC surface possesses a high Pb(II) adsorption capacity. The Langmuir model provided the best fit for the adsorption data, indicating monolayer coverage [37], with a calculated maximum adsorption capacity of 148.25 mg·g^−1^ (Figure 1). Furthermore, XPS analysis revealed that Pb(II) adsorption by NBC is a multi-mechanism process. These mechanisms include surface coordination between Pb(II) and oxygen- and nitrogen-containing functional groups (e.g., hydroxyl and pyrrolic N), alongside the precipitation of Pb(II) into inorganic phases. This synergistic combination of chemisorption and precipitation ensures efficient and stable immobilization of Pb(II).

### 4.2. NBC Enhanced Iron Plaque Formation and Reduced Pb Accumulation in Roots

Within the rhizosphere, iron is reduced to soluble Fe(II) via processes mediated by root exudates and microorganisms. This Fe(II) is subsequently oxidized by oxygen released from aerenchyma tissues through radial oxygen loss (ROL) [49]. The resulting precipitation of iron oxides forms an iron plaque on the root surface, which mitigates heavy metal toxicity by adsorbing metallic contaminants and protecting the plant [50].

Previous research on the effects of biochar on iron plaque formation in rice has yielded conflicting results; while some studies report that biochar promotes plaque development [51,52,53], others have observed inhibitory effects [54,55]. These discrepancies may be attributed to two primary factors: biochar application rates and inherent physicochemical properties. For instance, Gu et al. [56] demonstrated that low concentrations of nano-biochar significantly enhanced iron plaque formation by facilitating the direct oxidation of Fe(II) and electron transfer, whereas high concentrations impeded this process. Furthermore, the redox activity of biochar, governed by its redox-active functional groups and graphitic structures, is a critical determinant of its impact on iron plaque formation [56,57]. Functioning as an electron shuttle, biochar facilitates the microbial reduction of Fe(III), thereby enhancing iron plaque formation [52]. In the present study, the application of BC had no significant effect on iron plaque formation. In contrast, NBC addition significantly increased iron plaque formation by 21.98% to 26.19% compared to the control. This enhancement is likely attributable to the specific surface area of NBC, which is 6.30 times greater than that of BC (Table S2), providing more reactive sites and facilitating electron exchange. Furthermore, the NBC application significantly decreased the soil Eh (Figure 2) and increased the relative abundance of *Symbiobacteraceae* (Figure 6). These changes likely contributed to the enhanced reduction of Fe(III) in the rhizosphere [58], ultimately promoting iron plaque formation. Consequently, Pb concentrations in rice roots decreased significantly as iron plaque formation increased (Figure 5). This aligns with previous research demonstrating that iron plaque acts as a robust barrier, inhibiting the uptake of heavy metals into plant tissues [59,60].

### 4.3. NBC Enhanced Soil Fertility and Bacterial Diversity

Compared to the control, the application of NBC significantly increased soil pH, EC, SOM, SAN, SAK, and SAP (Figure 2). Given its inherent nutrient content, NBC directly enhances soil fertility; for instance, NBC application increased SAN content by 45.04–46.91% relative to the control. Previous studies have indicated that conventional nitrogen fertilization can reduce soil pH, decrease iron plaque formation on roots, and consequently increase heavy metal bioavailability [61]. In contrast, NBC exhibited the opposite effect: it not only increased soil nitrogen content but also promoted the formation of iron plaque on root surfaces, thereby facilitating the immobilization of soil Pb. Furthermore, the NBC application altered the soil microbial community structure, which subsequently influenced soil nutrient availability.

Microorganisms play a pivotal role in mediating fundamental soil processes, including the stabilization of soil structure, the decomposition of organic matter, and the cycling of nutrients [27,62,63]. In this study, the NBC application significantly increased the number of OUTs, as well as the ACE, Chao1, and Shannon indices, indicating a pronounced enhancement in the bacterial community richness and diversity. Rhizosphere microbial diversity and composition were strongly associated with soil nutrient content and Pb bioavailability (Figure 7a). Additionally, random forest (RF) analysis identified the acid-soluble Pb fraction as the most important predictor for OUTs, followed by SOM (Figure 7c). This is likely because acid-soluble Pb is highly bioavailable to plants and microorganisms, thereby exerting strong selective pressure on the microbial community and posing a significant ecological risk. Furthermore, an increase in SOM correlated with a significant rise in bacterial abundance, richness, and Shannon diversity [62,64]. SOM has also been shown to foster positive interactions between beneficial nematodes and soil microorganisms [62].

The dominant bacterial phyla in the soil included *Acidobacteriota*, *Proteobacteria*, *Firmicutes*, *Bacteroidota*, and *Gemmatimonadota*. *Proteobacteria* are widely recognized as a dominant phylum in heavy metal-contaminated soils due to their ability to resist metal(loid) toxicity through complexation and adsorption [65]. Following the NBC addition, the relative abundance of *Proteobacteria* increased, likely due to the improved availability of nutrients and organic matter [48,66]. Furthermore, the NBC application increased the abundance of *Chloroflexi*, a bacterial phylum known to enhance plant photosynthetic efficiency [67,68]. At the family level, *Comamonadaceae*, *Oxalobacteraceae*, *Bacillaceae*, and *Chitinophagaceae* were the predominant taxa. The relative abundance of *Bacillaceae*, *Comamonadaceae*, and *Nitrosomonadaceae* was significantly higher in BC2 and NBC2 treatments compared to the control (Figure 6). Members of the *Bacillaceae* family play fundamental roles in organic matter cycling and the promotion of plant health and growth [69,70]. *Nitrosomonadaceae* is recognized as a key contributor to the primary nitrogen cycling process in the soil [71,72], while *Comamonadaceae* is known to enhance nutrient cycling, degrade plant residues, and improve metal tolerance [73,74]. Collectively, these findings indicate that NBC addition effectively enriches beneficial bacterial populations in the soil.

Furthermore, NBC treatment resulted in a higher relative abundance of the bacterial families *Hungateiclostridiaceae* and *Symbiobacteraceae* compared to the BC treatment. Members of *Hungateiclostridiaceae* are actively involved in microbial sulfur reduction [75,76]. Meanwhile, the family *Symbiobacteraceae* comprises anaerobic, chemoorganotrophic bacteria that utilize sugars with nitrate as a terminal electron acceptor or rely on peptone as a sole substrate [58,77,78]. These specific shifts in the microbial community likely promoted the generation of soil sulfides, which subsequently facilitated the precipitation of Pb into insoluble forms.

However, several limitations of this study should be acknowledged. First, the preparation of NBC involves both ball-milling and N-doping, processes that concurrently alter particle size, surface area, and surface chemistry. Consequently, the enhanced performance of NBC relative to pristine biochar results from the synergistic effects of these modifications rather than the isolated impact of N-doping. Second, although Pb immobilization was partly attributed to sulfide precipitation, inferred from the increased abundance of *Hungateiclostridiaceae*, soil sulfide concentrations were not directly quantified. Third, because these findings are derived from a single soil type under controlled pot conditions, field validation across diverse soil environments is necessary. Finally, from an applied perspective, the 2.5–5% application rate is 62.5–125 t·ha^−1^ under field conditions. This poses potential economic and N-loading challenges that require further optimization. Future field-scale studies should address these limitations and optimize application strategies across various crops.

## 5. Conclusions

This study demonstrates that NBC is a highly effective amendment for the immobilization of Pb in contaminated soils. The remediation mechanism is driven by a combination of physicochemical and biological factors. First, NBC exhibits a high direct adsorption capacity for Pb(II). Second, its application significantly increases soil pore water pH and reduces redox potential (Eh), promoting Pb stabilization. Third, NBC enriches the relative abundance of the soil bacterial family *Hungateiclostridiaceae*, which likely facilitates soil sulfide generation and the subsequent precipitation of Pb into insoluble forms. Furthermore, the NBC application stimulates the formation of iron plaque on rice roots, thereby reducing Pb accumulation in the plants. Beyond direct remediation, NBC improves overall soil health by increasing SOM and nutrient (N, P, and K) availability. It also significantly enhances soil microbial diversity and richness, specifically promoting the abundance of key bacterial phyla (e.g., *Proteobacteria*, *Firmicutes*, *Actinobacteriota*) associated with robust soil ecosystems. Therefore, this work provides a mechanistic foundation for employing functionalized biochar as a sustainable remediation strategy. Future studies should systematically optimize NBC application rates and validate its efficacy across different crop species to facilitate field-scale application.

## Data Availability

The original contributions presented in this study are included in the article/Supplementary Materials. Further inquiries can be directed to the corresponding authors.

## Supplementary Materials

The following supporting information can be downloaded at: https://www.mdpi.com/article/10.3390/toxics14070561/s1, Table S1. Physicochemical properties of the tested soil; Table S2. Basic properties of the biochars; Table S3. Kinetic parameters of adsorption on Pb^2+^; Table S4. Parameters of Langmuir and Freundlich isotherm equation fitting for Pb^2+^; Figure S1. Adsorption kinetics of NBC for Pb(II) adsorption by fitting the pseudo-first-order and pseudo-second-order (a). Adsorption isotherms for Pb(II) on NBC by fitting the Langmuir and Freundlich models (b).
