# Supplementary material for: Nitrogen-Doped Straw Biochar Reduces Lead Toxicity in Paddy Rhizosphere Soil Through Physicochemical and Microbial Synergies
Source: Toxics. 2026 Jun 26;14(7):561. doi: 10.3390/toxics14070561 (PMC13417415; doi:10.3390/toxics14070561)
Supplement: Supplementary file 1 [file toxics-14-00561-s001.zip › toxics-4348555-supplementary.pdf]

Nitrogen-Doped Straw Biochar Reduces Lead Toxicity in Paddy Rhizosphere Soil Through Physicochemical and Microbial Synergies

Honghong Li [1,*], Zeyu Liu [1], Zhou Li [2,*], Chunle Chen [3] and Meiya Wang [1]

1 School of History and Geography, Minnan Normal University, Zhangzhou 363000, China

2 Institute of Subtropical Agriculture, Fujian Academy of Agricultural Sciences, Zhangzhou 363005, China

3 School of Resources and Chemical Engineering, Sanming University, Sanming 365004, China

* Correspondence: lhh1819@mnnu.edu.cn (H.L.); lizhou@faas.cn (Z.L.)

Table supplementary captions:

Table S1. Physicochemical properties of the tested soil.

Parameters	value
pH	6.50
CEC (cmol(+)·kg^{-1})	12.59
Organic matter (g·kg^{-1})	22.0
Metals (mg·kg-1)	
Cd	6.28
Pb	2640
Zn	1480
Soil texture	Sandy clay loam
Particle size distribution (%)	
Sand	64
Silt	20.4
Clay	15.6

Table S2. Basic properties of the biochars.

Biochars	Elemental composition					Atomic ratio			SBET
	C（%）	N（%）	H（%）	O（%）	S（%）	H/C	O/C	(O+N)/C	(m^2·g^{-1})
BC	38.82	1.33	1.48	6.83	1.5	0.038	0.18	0.21	9.03
N-BC	38.54	1.49	1.43	6.24	1.25	0.037	0.16	0.20	56.85

Table S3. Kinetic parameters of adsorption on Pb^{2+}.

	pseudo-first-order			pseudo-second-order		
	q_e (mg·g^{-1})	k_1 (1·min^{-1})	R^2	q_e (mg·g^{-1})	k_2 (g·mg^{-1}·min^{-1})	R^2
N-BC	93.74	0.0157	0.8674	102.07	0.00020	0.9524

Table S4. Paramerers of Langmuir and Freundlich isotherm equation fitting for Pb^{2+}.

	Langmuir model			Freundlich model		
	q_m (mg·g^{-1})	K_L (L·mg^{-1})	R^2	K_F (mg·g^{-1})(L·mg^{-1})$^{1/n}$	1/n	R2
N-BC	148.25	0.0345	0.9838	23.72	0.3086	0.9393

Figure supplementary captions:

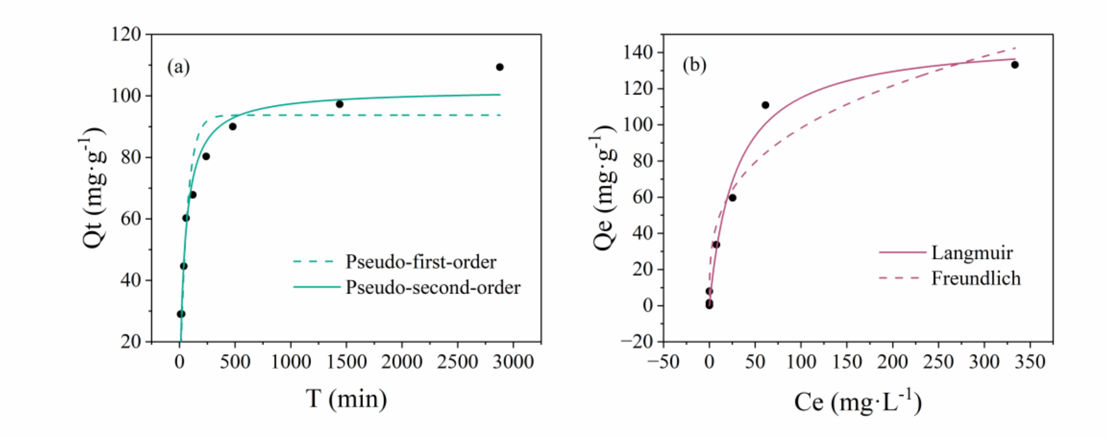

Figure S1. Adsorption kinetics of NBC for Pb(II) adsorption by fitting the pseudo-first-order and pseudo-second-order (**a**). Adsorption isotherms for Pb(II) on NBC by fitting Langmuir and Freundlich model (**b**).
